# The PLAGL2/MYCN/miR-506-3p interplay regulates neuroblastoma cell fate and associates with neuroblastoma progression

**DOI:** 10.1186/s13046-020-1531-2

**Published:** 2020-02-22

**Authors:** Zhenze Zhao, Spencer D. Shelton, Alejandro Oviedo, Amy L. Baker, Collin P. Bryant, Soroush Omidvarnia, Liqin Du

**Affiliations:** Department of Chemistry and Biochemistry, Texas State University, 601 University Drive, San Marcos, TX 78666 USA

**Keywords:** miR-506-3p, Neuroblastoma, PLAGL2, MYCN, Retinoic acid, Cell differentiation

## Abstract

**Background:**

The oncogene MYCN is critical for tumorigenesis of several types of cancers including neuroblastoma. We previously reported that miR-506-3p repressed MYCN expression in neuroblastoma cells. However, the mechanism underlying such regulation was undetermined since there is no miR-506-3p target site in MYCN 3’UTR.

**Methods:**

By a systematic investigation combining microarray, informatics and luciferase reporter assay, we identified that the transcriptional factor pleiomorphic adenoma gene-like 2 (PLAGL2) is a direct target of miR-506-3p that mediates its regulation on MYCN expression. Using CHIP-PCR and luciferase reporter assay, we validated the transcriptional regulation of MYCN by PLAGL2 and we further demonstrated the transcriptional regulation of PLAGL2 by MYCN. We examined the function of PLAGL2 in regulating neuroblastoma cell fate by cell viability assay, colony formation and Western blotting of differentiation markers. We examined the effect of retinoic acid, the differentiation agent used in neuroblastoma therapy, on miR-506-3p, PLAGL2 and MYCN expressions by quantitative PCR and Western blots. We investigated the clinical relevance of PLAGL2 expression by examining the correlation of tumor PLAGL2 mRNA levels with MYCN mRNA expression and patient survival using public neuroblastoma patient datasets.

**Results:**

We found that miR-506-3p directly down-regulated PLAGL2 expression, and we validated a PLAGL2 binding site in the MYCN promoter region responsible for promoting MYCN transcription, thereby establishing a mechanism through which miR-506-3p regulates MYCN expression. Conversely, we discovered that MYCN regulated PLAGL2 transcription through five N-Myc-binding E-boxes in the PLAGL2 promoter region. We further confirmed the reciprocal regulation between endogenous PLAGL2 and MYCN in multiple neuroblastoma cell lines. Moreover, we found that PLAGL2 knockdown induced neuroblastoma cell differentiation and reduced cell proliferation, and combined knockdown of PLAGL2 and MYCN showed a synergistic effect. More strikingly, we found that high tumor PLAGL2 mRNA levels were significantly correlated with high MYCN mRNA levels and poor patient survival in neuroblastoma patients. Furthermore, we found that retinoic acid increased expression of miR-506-3p and repressed expression of MYCN and PLAGL2.

**Conclusions:**

Our findings altogether suggest that the interplay network formed by PLAGL2, MYCN and miR-506-3p is an important mechanism in regulating neuroblastoma cell fate, determining neuroblastoma prognosis, and mediating the therapeutic function of retinoic acid.

## Background

It is well known that aberrant expression of the MYCN gene, which encodes the transcription factor (TF) N-Myc, plays a key role in neuroblastoma tumorigenesis [1–6]. One of the well-characterized mechanisms that lead to MYCN overexpression in neuroblastoma is MYCN gene amplification, which is observed in 25% of neuroblastomas and is associated with poor patient prognosis [3, 7]. Experimental demonstration for the critical role of MYCN in neuroblastoma was from MYCN transgenic mouse models in which neuroblastoma spontaneously developed [8]. The above evidence supports that blocking MYCN expression would be beneficial for neuroblastoma patients. However, N-Myc protein currently remains largely un-druggable as its active DNA-binding domain is composed of two extended alpha helices with no apparent surfaces for small molecule binding [9]. The mechanisms that drive MYCN overexpression at transcriptional and post-transcriptional levels have been recognized. These levels of regulatory mechanisms possibly provide paths to target the MYCN pathway for therapy, if druggable molecules are identified in its regulatory network.

One of the regulatory mechanisms of MYCN expression is found at the transcription level. For example, E2F and Sp1/Sp3 binding sites were identified in the MYCN promoter, and the activation of the MYCN promoter required cooperative binding of these TFs to the promoter [10, 11]. However, E2F and Sp1/Sp3 were not sufficient to activate the MYCN gene expression [11], indicating additional requirements for its transcriptional activation. Additional transcription mechanisms have been investigated [12–15]. One such mechanism was demonstrated by Schonnerr et al., showing that anaplastic lymphoma kinase (ALK) was involved in initiating the transcription of MYCN in neuroblastoma cells [15], providing a potential therapeutic path to block the oncogenic function of MYCN. However, elevated ALK activity occurs only in around 14% of high-risk neuroblastoma [16], suggesting that MYCN is not targetable through the ALK pathway in the majority of neuroblastoma cases. Overall, even though the knowledge on the mechanisms of MYCN transcriptional regulation has been advanced in recent years, the regulatory complex of MYCN transcription is far from being fully defined. Further elucidation of its transcription mechanisms would benefit the development of novel approaches to target the MYCN pathway.

Another class of regulators of MYCN expression is microRNAs (miRNAs). Multiple miRNAs have been identified to regulate MYCN expression either by directly targeting the 3′ untranslated region (3’UTR) of MYCN mRNA or through indirect pathways [17–19]. Our group previously identified miR-506-3p as a potent differentiation inducer and a strong repressor of MYCN expression in neuroblastoma cells [17, 20]. However, MYCN is not a direct target of miR-506-3p and the mechanism of its regulation by miR-506-3p was not determined in the previous study [17]. In this study, we demonstrate that miR-506-3p directly targets the 3’UTR of Pleiomorphic adenoma gene like-2 (PLAGL2), and the latter functions as an activator of MYCN transcription through a PLAGL2 binding site in the MYCN promoter, unraveling the mechanism by which miR-506-3p regulates MYCN expression.

On the other hand, N-Myc is known to regulate transcription of many genes involved in a variety of biological processes including cell differentiation [21, 22]. Here we investigated the transcriptional regulation of PLAGL2 by N-Myc in neuroblastoma cells. Furthermore, cell differentiation has been well known to play a key role in neuroblastoma tumorigenesis and differentiation therapy [3, 23]. Neuroblastoma cell differentiation is triggered by certain signals, including repression of MYCN expression [4, 24, 25], overexpression of tumor suppressive miRNAs [20, 26, 27], and pharmacological concentrations of differentiation agent retinoic acid (RA) [28]. Therefore, following the discovery of the interplay between PLAGL2, MYCN and miR-506-3p, we further investigated the function of PLAGL2 in regulating neuroblastoma cell differentiation and the possible involvement of this interplay network in mediating the cellular function of RA, and investigated the clinical relevance of PLAGL2 in determining neuroblastoma prognosis.

## Materials and methods

### Cell lines and materials

The human neuroblastoma cell lines BE(2)-C, SKNDZ, IMR322, SKNFI, SKNBE [2], CHP212, SKNMC, SKNAS, MCIXC, and SKNSH were obtained from American Type Culture Collection (Manassas, VA, USA); CHLA-90 and COG-N-322 (N322) were from Children’s Oncology Group (Monrovia, CA, USA); KELLY and NGP were from the cell line repository at the Greehey Children’s Cancer Research Institute at the University of Texas Health San Antonio. HEK 293T cells were from Thermo Fisher Scientific (Hampton, NH, USA). Cells were cultured in DMEM/F12 (Corning Inc., Corning, NY, USA) with 10% Equafetal bovine serum (Atlas Biologicals, Fort County, CO, USA). miR-506-3p mimic, siRNAs and negative control oligos were purchased from Dharmacon (Lafayette, CO, USA). PCR Primers were designed in-house (sequences are listed in Additional file 1: Table S1) and purchased from Sigma-Aldrich (St. Louis, MO, USA). Rabbit anti-N-Myc (α-N-Myc), anti-PCNA, anti-Ki67, anti-cleaved PARP, anti-calnexin, and HRP-conjugated anti-rabbit IgG antibodies were from Cell Signaling Technology (Danvers, MA, USA). Rabbit anti-PLAGL2 (α-PLAGL2) antibody was from MyBioSource (San Diego, CA, USA). Rabbit anti-βIII tubulin, anti-GAP43, and anti-NSE were from Abcam (Cambridge, UK). PLAGL2 expression vector pCMV3-PLAGL2 (Cat. NO: HG20912-UT) was from Sino Biological (Wayne, PA, USA). MYCN expression vector pCMV-XL4-MYCN (Cat. NO: SC116780) was from Origene (Rockville, MD, USA). All-*trans* RA (ATRA) and 13-*cis*-RA were purchased from Sigma-Aldrich.

### Cell viability and proliferation assay

Cell viability and proliferation assays were used to measure the effect of different treatments on cell survival and proliferation. Cell viability was measured by MTT (2-(4,5—Dimethylthiazol-2-yl)-2,5-Diphenyltetrazolium Bromide) assay as previously described [29]. For measuring cell proliferation, 2500 cells were plated and treated in 96-well plates. Cell images were taken every 24 h under 20X magnification in Incucyte ZOOM Live Cell Imaging System (Essen Bioscience, Ann Arbor, MI, USA) and cell confluence was measured based on the collected cell images. Cell proliferation was determined by measuring increase in cell confluence over time.

### Detection of neurite outgrowth

2500 cells were plated and treated as specified in 96-well plates. For measuring neurite outgrowth, cell images were taken under 20X magnification in an IncuCyte ZOOM Live Cell Imaging System (Essen BioScience), and relative neurite length was calculated as described previously [20].

### Colony formation assay

Colony formation assay was used to measure the long-term effect of treatments on cell proliferation, and it was performed as previously described [29]. Colony numbers and sizes were quantified using Image J (NIH, Bethesda, MD). Specifically, the scanned images were first converted to greyscale, and then the area to be analyzed was highlighted and a binary image was created. The colonies were displayed as black particles in the binary image and the number of colonies was automatically counted. To analyze the colony size, the total area occupied by all the colony particles was automatically counted, and the average size of the colonies was calculated via dividing the total colony area by the number of colonies.

### Quantitative polymerase chain reaction (qPCR)

Total RNA was isolated using Trizol reagent. 2 μg RNA was reverse transcribed using Superscript II and random primers (Applied Biosystems, Waltham, MA, USA). An aliquot of cDNA corresponding to 50 ng of RNA was used for running SYBR Green-based qPCR analysis using ABI 7000.

### Western blots

Cell lysate collection, SDS-PAGE gel electrophoresis, transfer to PVDF membranes, and Western blotting were performed as previously described [29]. For quantitative comparison between treatment groups, the raw band intensities in the Western blot images were quantified using Image J, and the relative band intensities were derived as the following: the raw band intensity of a specific protein in a specific treatment group was first normalized to its corresponding loading control (i.e., calnexin). Then the calnexin-normalized band intensity in the treatment group was further normalized to that in the control group. For example, for analyzing the effect of miR-506-3p mimic on N-Myc protein expression, the raw N-Myc band intensity in each treatment group was first normalized to the raw band intensity of the calnexin blot in the corresponding treatment group, and then the calnexin-normalized N-Myc band intensity in the miR-506-3p mimic treatment group were further normalized to that in the control oligo group to obtain the final relative intensity.

### Chromatin immunoprecipitation-PCR (CHIP-PCR)

CHIP-PCR was used to validate the binding of PLAGL2 and MYCN proteins to their predicted binding sites in the corresponding promoter regions. CHIP was performed according to published procedures with modifications [10]. Briefly, four 10-cm dishes of sub-confluent cells were cross-linked with 1% formaldehyde. Cells were collected in lysis buffer (5 mM Tris, pH 8, 85 mM KCl, 0.5% NP-40, and protease inhibitors) and ruptured by passing through a 22-gauge needle ten times. Nuclei were then pelleted and re-suspended in sonication buffer (10 mM Tris pH 7.5, 150 mM NaCl, 1 mM EDTA, 1% NP-40, 1% deoxycholate, 0.1% SDS, and protease inhibitors). After incubation on ice for 10 min, the solution was sonicated with a Tissue Tearor Homogenizer (Cole-Parmer, Cernon Hills, MI, USA) at half maximum power for ten 10-s pulses on ice. The chromatin solution was centrifuged, and the supernatant was then pre-cleared with protein A/G agarose beads (Santa Cruz Biotech, Santa Cruz, CA, USA). Aliquots of the pre-cleared chromatin were incubated with 2 μg antibody overnight at 4 °C. One aliquot (100 μl) was incubated without antibody (this sample is referred to as Input). Immune complexes were captured with 40 μl of BSA-blocked protein A/G agarose, and then digested with RNase A and proteinase K. The co-precipitated DNA was isolated using PCR purification column (Qiagen, Germantown, MD, USA). 1 μl of the isolated DNA was used for PCR.

### pmiRGLO 3’UTR luciferase reporter assay

The pmiRGLO 3’UTR Luciferase reporter assay was used to validate the target site of miR-506-3p in the 3’UTR in the PLAGL2 mRNA. To generate the wildtype luciferase reporter (PLAGL2-WT) for the predicted miR-506-3p target site in the 3’UTR of PLAGL2, a fragment of the PLAGL2 3’UTR (3008 bp to 3671 bp) containing the predicted target site (UGCCUUA) of miR-506-3p was amplified from human genomic DNA by PCR using Phusion enzyme (New England Biolabs, Ipswich, MA, USA) and inserted downstream of firefly luciferase coding sequence (CDS) in the pmirGLO dual-luciferase reporter (Promega, Madison, WI, USA) using In-Fusion cloning kit (Takara Bio USA, Mountain View, CA, USA). Mutant reporter (PLAGL2-MU) with mutated seed sequence (CCUCAGA) at predicted target site was generated by site-directed mutagenesis using high fidelity DNA polymerase (Agilent Technologies, Santa Clara, CA, USA). BE(2)-C cells were co-transfected with pmiRGLO luciferase reporter and miR-506-3p for 2 days, and luciferase activity was measured using the Dual-Luciferase Reporter Assay System (Promega) in a BioTek Synergy H4 microplate reader.

### pGL3B promoter luciferase reporter assay

The pGL3B promoter luciferase reporter assay was used to validate the function of the PLAGL2- and MYCN-binding DNA sequences in regulating gene transcription. The putative PLAGL2 binding site in the MYCN gene promoter region and the N-Myc-binding E-boxes in the PLAGL2 gene promoter region were predicted using ConTra V3 prediction tool. A DNA fragment from the wildtype MYCN (− 480 bp to − 124 bp) promoter region containing the putative PLAGL2 binding site (CCCCCGGAGCCCTC) was amplified and inserted upstream of firefly luciferase gene in the pGL3B reporter vector (Promega) between Sac I and Hind III cloning sites to generate the wildtype reporter (mycP_WT_-Luc). Mutant pGL3B reporter (mycP_MU_-Luc) containing mutated binding site (CCTCAGCTCCACTC) was generated as above. For the N-Myc-binding E-boxes in PLAGL2 gene promoter, DNA fragments from the PLAGL2 promoter region containing different combinations of the 5 putative E-boxes were amplified and inserted into pGL3B luciferase reporter to generate three reporter vectors, plaP_(E1–5)_-Luc reporter which contains all five E-boxes (− 640 bp to − 28 bp), plaP_(E1–3)_-Luc which contains E1 to E3 E-boxes (− 445 bp to − 28 bp), and plaP_(E4–5)_-Luc which contains E4 and E5 E-boxes (− 640 bp to − 429 bp). A reporter without an insert was used as a negative control. 293T cells were co-transfected with either PLAGL2 or MYCN expression vector and the corresponding luciferase reporter at 1:1 mole ratio. Luciferase activities were measured using the Luciferase Assay System (Promega) after 48 h.

### mRNA expression array

mRNA expression array was conducted to examine the effect of miR-506-3p overexpression on gene expression as previously reported [20]. Total RNA was isolated using the miRVana miRNA isolation kit (Life Technologies, Carlsbad, CA, USA) as previously reported [30]. mRNA expression profiling was performed using the Illumina mRNA WG-6 v3 microarray platform as previously reported [20].

### Neuroblastoma patient survival analysis and tumor gene expression correlation analysis

The analyses were performed to examine the clinical relevance of PLAGL2 expression in neuroblastoma tumors, and were conducted using three published neuroblastoma patient datasets in the R2: Genomics Analysis and Visualization Platform [31], the Kocak (649 patients), SEQC (498 patients) and NRC (283 patients) datasets. Patient survival analyses were conducted using the Kaplan-Meier method offered by the R2 platform. In detail, the neuroblastoma patients were first divided into two groups based on the analysis of the overall patient survival by using the Kaplan Scan (Scan) cutoff modus offered by the R2 program, where an optimum survival cutoff was established based on statistical testing. Specifically, in the order of the patient tumor PLAGL2 mRNA expression, the Scan module uses every increasing expression value as a cutoff to create two groups (i.e., the high and low PLAGL2 mRNA groups) and test the *p*-value in a log rank test, and then the most significant expression cutoff for the overall survival analysis is identified. The overall and event-free Kaplan-Meier survival curves for the two identified groups are then derived. The statistical significance of the difference in PLAGL2 mRNA levels between the two groups were examined by unpaired t-test, with *p* < 0.05 considered as statistically significant. The statistical significance of difference in survival between groups was determined by 2-tailed log-rank test with *p* < 0.05 considered statistically significant. Correlations between MYCN mRNA levels and PLAGL2 mRNA levels were assessed by Pearson correlation, with *p* < 0.05 considered statistically significant.

### Other statistical analyses

To evaluate the effect of treatments, the statistical significance of difference between each treatment and the control group was determined by two-tailed Student’s *t*-test, with *p* < 0.05 considered statistically significant. The synergistic effect of combined treatment on cell viability was evaluated based on the Bliss Independence model, which defines the predicted additive effects of the two treatment groups by E_xy_ = E_x_ + E_y_ – E_x_E_y_, where E_x_ and E_y_ are the effects of the individual treatments of X and Y on cell viability [32].

## Results

### miR-506-3p has a generic effect in down-regulating MYCN expression in neuroblastoma cell lines

**Fig. 1:**
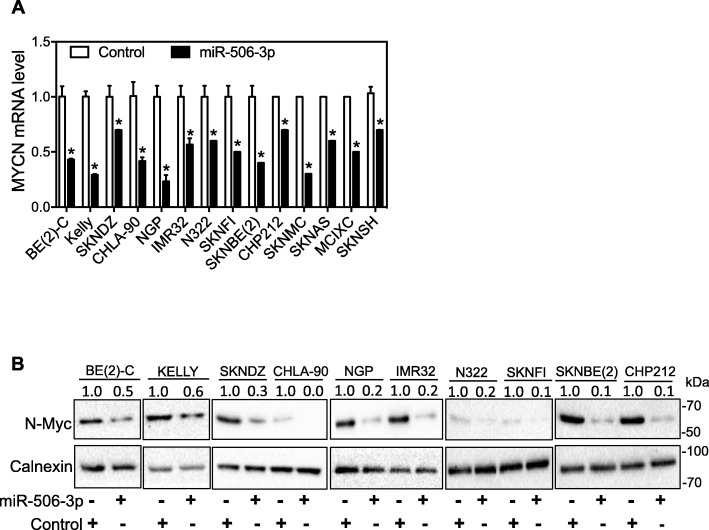
miR-506-3p down-regulated MYCN expression. Cells were transfected with 25 nM miR-506-3p mimic or control oligo for 2 days, and RNAs and protein lysates were harvested. **a** MYCN mRNA levels as measured by qPCR. Shown are the relative expression level of MYCN mRNA in each cell line transfected with the miR-506-3p mimic normalized to that in the same cell line transfected with control oligo. *, *p* < 0.05 comparing to control. **b** Representative Western blots of N-Myc protein levels, with Calnexin protein levels measured as a loading control. Values shown above the N-Myc bands are the calculated relative intensities of the corresponding bands

We previously found that miR-506-3p down-regulated MYCN expression in three neuroblastoma cell lines [17]. Here we further investigated the function of miR-506-3p in regulating MYCN expression in additional neuroblastoma cell lines with distinct genetic backgrounds (Additional file 2: Table S2). As shown in Fig. 1a, miR-506-3p overexpression significantly down-regulated MYCN expression at mRNA levels in all fourteen cell lines. Figure 1b further shows that miR-506-3p dramatically down-regulated N-Myc protein expression in ten cell lines. Although the N-Myc levels were not detectable in four MYCN non-amplified cell lines (SKNMC, SKNAS, MCIXC and SKNSH) using currently available antibodies, results in Fig. 1a clearly show that miR-506-3p effectively down-regulated MYCN expression at mRNA levels in these cells. These results confirm that miR-506-3p mimic has a generic effect in down-regulating MYCN expression in neuroblastoma cells regardless of the MYCN amplification status and other genetic backgrounds.

### PLAGL2 is identified as a direct target of miR-506-3p that regulates transcription of MYCN gene

**Fig. 2:**
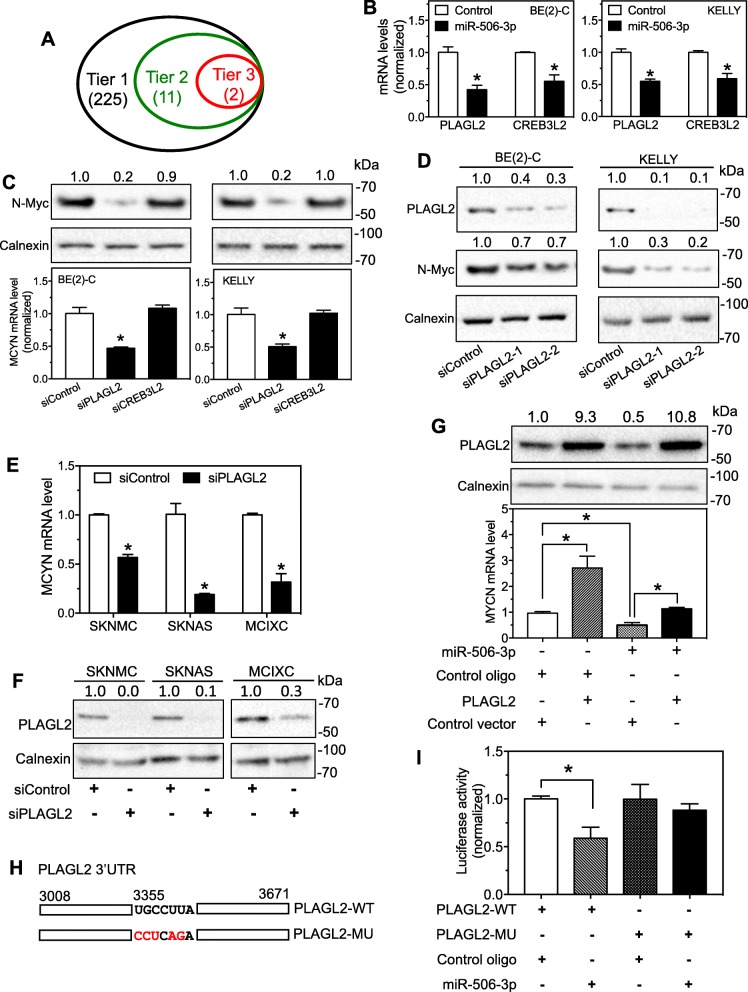
Identification of PLAGL2 as the miR-506-3p target that regulates MYCN expression. **a** Venn diagram showing the strategy to identify TFs that are predicted to be direct targets of miR-506-3p and are also predicted to regulate MYCN transcription. The numbers of genes identified are shown in parentheses. Tier 1 identifies 225 genes that were down-regulated by ≥40% by miR-506-3p mimic. Tier 2 identifies 11 TFs from the 225 genes. Tier 3 identifies 2 TFs that are predicted to be direct targets of miR-506-3p. **b** Effect of miR-506-3p overexpression on the mRNA expression of the two TFs in BE(2)-C and Kelly cells. Cells were transfected with 25 nM miR-506-3p mimic or control oligo for 2 days. PLAGL2 and CREB3L2 mRNAs were detected by qPCR. **c** Effect of knocking down the expression of the two TFs on MYCN expression. Cells were transfected with the indicated siRNAs or control oligo (siControl) at 25 nM for 2 days. MYCN mRNA and N-Myc were detected by qPCR and Western blots, respectively. **d** Effect of additional siPLAGL2s on N-Myc expression. Cells were transfected with the indicated oligos (25 nM) and protein levels were measured as above. **e-f** Effect of PLAGL2 knockdown on MYCN mRNA expression in three MYCN non-amplified neuroblastoma cell lines. Cells were transfected with siPLAGL2 or siControl (25 nM) for 2 days, and MYCN mRNA levels were measured as above. **e**, mRNA expression as measured by qPCR. **f**, The depletion of PLAGL2 protein expression by the siPLAGL2 was confirmed by Western blots. **g** Effect of PLAGL2 overexpression on MYCN mRNA expression in SKNSH cells. Cells were transfected with the indicated vectors (PLAGL2 or Control, 0.8 ng/μl) and oligos (miR-506-3p or control oligo, 2.5 nM) for 2 days, and MYCN mRNA and PLAGL2 protein levels were measured as above. **h-i** Luciferase reporter assay for validating the target site of miR-506-3p in the 3’UTR of PLAGL2. **h**, Schematic graph of the cloned wildtype (PLAGL2-WT) and mutant (PLAGL2-MU) 3’UTR. The mutated nucleotides are shown in red. **i**, Validation of the target site by luciferase assay. BE(2)-C cells were co-transfected with the indicated vectors (0.8 ng/μl) and oligos (miR-506-3p mimic or control oligo, 2.5 nM). Two days after transfection, cells were lysed and luciferase activity was measured. *, *p* < 0.05. Values shown above the bands in the Western blot images are the calculated relative intensities of the corresponding bands

Since there is no miR-506-3p target site in the 3’UTR of MYCN, we speculate that miR-506-3p directly targets a TF that promotes MYCN expression. We combined expression microarray analysis with informatics analysis and literature search to systematically identify putative TFs that are down-regulated by miR-506-3p, predicted to be direct targets of miR-506-3p, and also predicted to transcriptionally regulate MYCN expression. As shown in Fig. 2a, we first exploited expression array to identify genes with their expression down-regulated by ≥40% following transfection of BE(2)-C cells with miR-506-3p mimic for 24 h (Tier 1), which yielded 225 genes. We then combined Ingenuity Pathway Analysis (IPA) and PubMed literature search to identify the TFs among the 225 genes (Tier 2, yielded 11 genes). Finally, we exploited the published TargetScan program [33] to identify TFs that are predicted to be direct targets of miR-506-3p (Tier 3), which yielded two TFs, PLAGL2 and CAMP Responsive Element Binding Protein 3 Like 2 (CREB3L2). Additional file 3: Table S3 and Additional file 4: Table S4 shows the predicted target sites of miR-506-3p in the 3’UTRs of the two TFs and the extent of down-regulation of their mRNAs by miR-506-3p detected in the expression microarray, respectively. We further confirmed the down-regulation of PLAGL2 and CREB3L2 mRNAs by miR-506-3p in independent experiments in two cell lines (Fig. 2b).

To determine whether PLAGL2 and CREB3L2 regulate MYCN expression, we transiently knocked down PLAGL2 and CREB3L2 expression using siRNAs in two cell lines. As shown in Fig. 2c, siPLAGL2 but not siCREB3L2 significantly decreased both MYCN mRNA and N-Myc protein levels. Two additional siRNAs designed to target different sites of the PLAGL2 transcript confirmed the decrease of N-Myc expression by PLAGL2 knockdown (Fig. 2d), excluding the possibility of off-target effect of siPLAGL2s on MYCN expression. We further examined the effect of PLAGL2 knockdown on MYCN expression at mRNA level in three MYCN non-amplified cell lines. As shown in Fig. 2e, siPLAGL2 significantly down-regulated MYCN mRNA levels in all three cell lines; the depletion of PLAGL2 protein by siPLAGL2 was confirmed in parallel (Fig. 2f). In addition, we showed that over-expression of PLAGL2 in a MYCN non-amplified cell line SKNSH increased MYCN mRNA levels and compromised the extent of MYCN down-regulation by miR-506-3p (Fig. 2g), although the up-regulation of MYCN expression by PLAGL2 was not detectable at protein level. These results altogether indicate that the regulation of MYCN expression by PLAGL2 is a generic mechanism in neuroblastoma cells.

To confirm the target site of miR-506-3p in the 3’UTR of PLAGL2 mRNA, wildtype (PLAGL2-WT) and mutated (PLAGL2-MU) luciferase reporters were constructed (Fig. 2h). As shown in Fig. 2i, miR-506-3p mimic significantly decreased luciferase activity in cells expressing PLAGL2-WT comparing to control oligo, whereas it did not significantly decrease luciferase activity in cells expressing PLAGL2-MU. These results indicate that the predicted target site of miR-506-3p in the 3’UTR of PLAGL2 truly mediates the down-regulation of PLAGL2 expression by miR-506-3p.

### PLAGL2 promotes MYCN transcription by directly binding to a specific sequence upstream of the CDS of MYCN gene

**Fig. 3:**
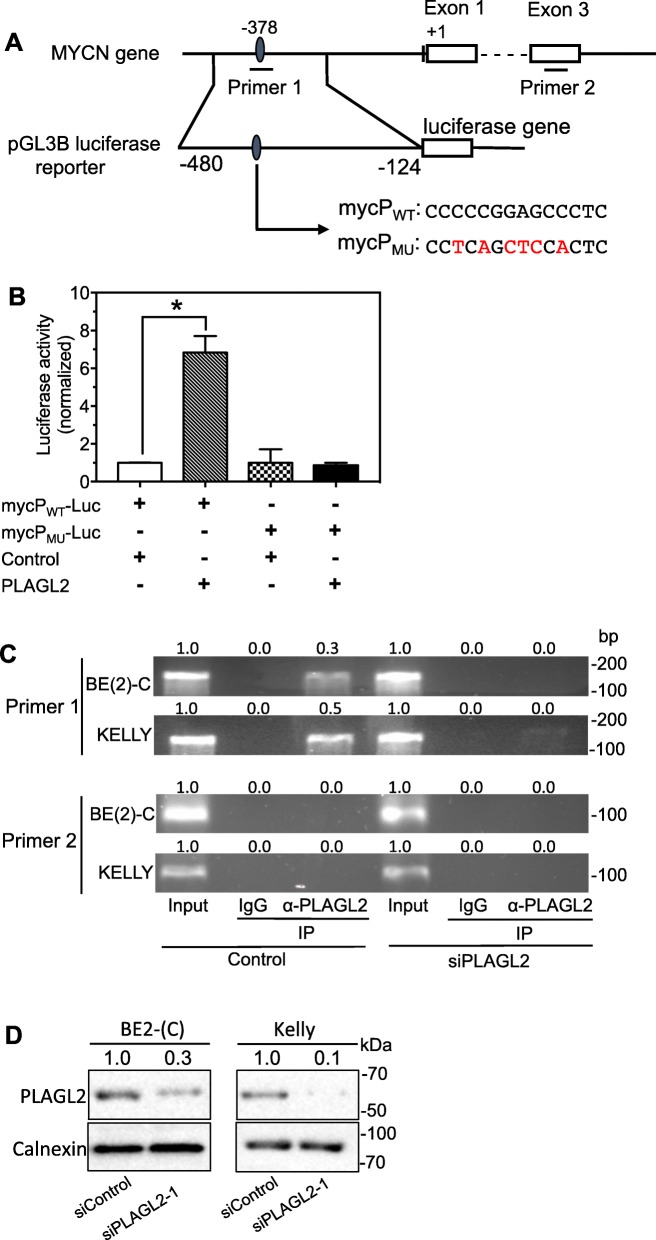
Validation of the PLAGL2 binding site in the MYCN promoter region. **a** Schematic diagram showing the putative PLAGL2 binding site in the MYCN promoter region and the region to be amplified to construct the luciferase reporter. The PLAGL2 binding site starts at − 378 nt. A segment of DNA (− 480 bp to − 124 bp) containing the predicted target site was amplified and inserted upstream of luciferase gene in pGL3B reporter to construct a wildtype luciferase reporter (mycP_WT_-Luc). A mutant vector with the binding site mutated was generated to use as a control (mycP_MU_-Luc, mutated nucleotides are shown in red). **b** Validation of the target site by luciferase assay in HEK 293T cells. Cells were co-transfected with the indicated luciferase reporters and PLAGL2 over-expression vector (or the Control vector) for 2 days, and luciferase activity was measured. *, *p* < 0.05. **c** Validation of the direct binding of PLAGL2 to its target site in the MYCN promoter by CHIP-PCR assay. CHIP were performed in two cell lines, BE(2)-C and KELLY. PCR was performed using the primer sets shown in (**a**). Shown are the representative PCR results under the indicated treatments. Values shown above the bands are the relative band intensities derived by normalizing the raw band intensities in the IP samples to those in the Input samples of the corresponding cells lines. **d** The depletion of PLAGL2 protein expression by the siPLAGL2 used in the CHIP-PCR experiment was confirmed by Western blots. Cells were transfected with the siPLAGL2 or control oligo (25 nM) for two days and protein levels were measured and quantified as above

We identified a putative PLAGL2 binding site at the 378-nucleotide position upstream of the MYCN CDS (Fig. 3a). To examine whether this is a functional regulatory site of PLAGL2, wildtype (mycP_WT_-Luc) and mutated (mycP_MU_-Luc) luciferase reporters were constructed (Fig. 3a). Each reporter was transfected into HEK 293T cells together with a PLAGL2 over-expression vector (PLAGL2) or a control expression vector (Control). As shown in Fig. 3b, PLAGL2 overexpression significantly increased luciferase activity in mycP_WT_-Luc cells, but not in mycP_MU_-Luc cells, comparing to their corresponding controls. These results indicate that the predicted binding site is a true target site of PLAGL2 that regulates transcription.

To further examine whether PLAGL2 directly binds to the above sequence, CHIP-PCR was performed in two cell lines. Figure 3a illustrates the positions of the DNA sequences to be amplified by each PCR primer sets, with primer 1 used to amplify the predicted PLAGL2-binding sequence and primer 2 used as a negative control. As shown in Fig. 3c, anti-PLAGL2 antibody (α-PLAGL2) successfully pulled down the predicted PLAGL2-binding sequence but not the control sequence. In addition, non-specific IgG did not pull down any of the above sequences, excluding the possibility of non-specific pulldown of protein-DNA complexes by IgGs. Furthermore, the predicted PLAGL2-binding sequence was not pulled down by α-PLAGL2 antibody in cells with PLAGL2 expression depleted by siPLAGL2, further supporting the specificity of the assay. The extent of PLAGL2 protein depletion by siPLAGL2 was confirmed by Western blots (Fig. 3d). Together, these results demonstrate that PLAGL2 activates MYCN transcription through directly binding to the identified PLAGL2-binding sequence in the promoter region of MYCN.

### N-Myc promotes PLAGL2 transcription through multiple binding sites in the PLAGL2 promoter region

**Fig. 4:**
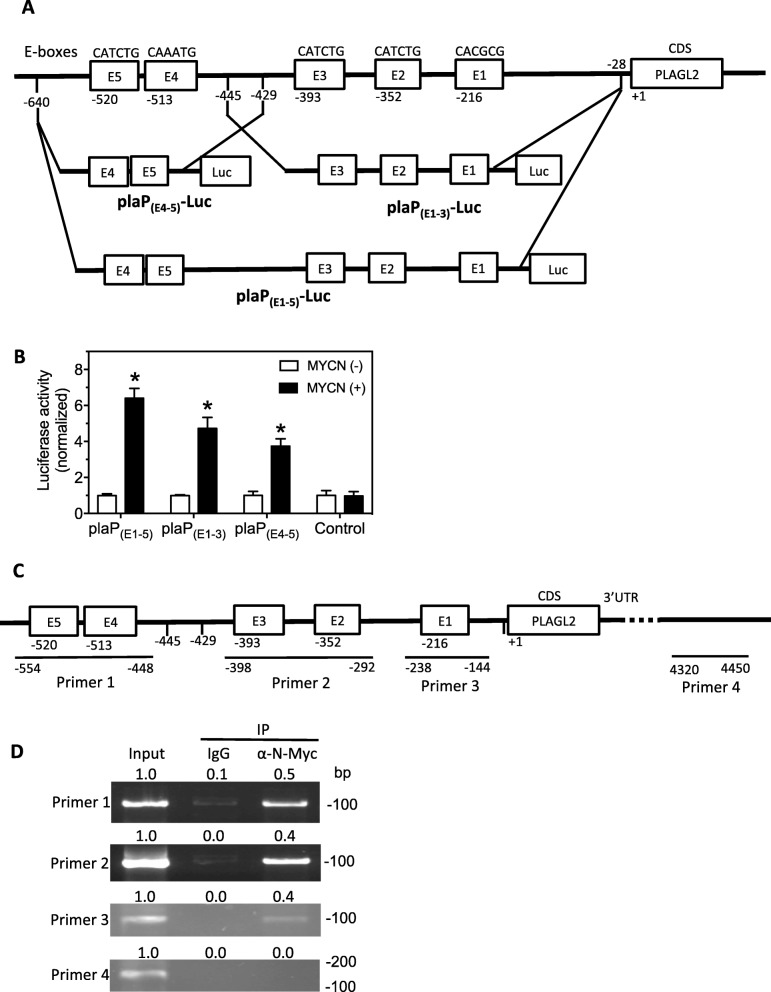
Validation of the N-Myc target sites in the PLAGL2 promoter region. **a** Schematic diagram showing the five putative N-Myc binding E-boxes (E1-E5) in the PLAGL2 gene promoter region and the amplified regions to construct the luciferase reporters. Three luciferase reporters, including plaP_(E1–5)_-Luc (spanning all 5 E-boxes), plaP_(E1–3)_-Luc (spanning E1 to E3) and plaP_(E4–5)_-Luc (spanning E4 and E5), were generated. **b** Validation of the target sites by luciferase assay in HEK 293T cells. A MYCN over-expression vector (MYCN(+)) or a control expression vector (MYCN(−)) was co-transfected with the indicated luciferase reporter or a Control reporter into cells. After 2 days, the luciferase activity was measured. *, *p* < 0.05. **c-d** Validation of the PLAGL2 target sites by CHIP-PCR assay. **c** The primers sets used in the assay. Primers 1, 2 and 3 were used to validate the E4-E5, E2-E3 and E1 sites, respectively. **d**, The CHIP-PCR results performed using the indicated primers sets under the indicated treatments. The relative band intensity shown above each band was normalized as above

Since N-Myc is known to regulate transcription of many genes, we were curious about its role in regulating PLAGL2 transcription. Remarkably, we found five putative N-Myc-binding E-boxes upstream of PLAGL2 CDS (Fig. 4a). To test whether these E-Boxes are true N-Myc binding sites, segments of DNA sequences containing the indicated E-Boxes were amplified to construct three reporters, the plaP_(E1–5)_-Luc, plaP_(E4–5)_-Luc and plaP_(E1–3)_-Luc (Fig. 4a). A reporter with no insertion was used as a negative control (Control). Each reporter was co-transfected with a MYCN overexpression vector (MYCN (+)) or a control vector (MYCN (−)). As shown in Fig. 4b, MYCN overexpression resulted in a significant increase of luciferase activity in cells expressing the plaP_(E1–5)_-Luc, plaP_(E4–5)_-Luc and plaP_(E1–3)_-Luc but not in cells expressing the Control reporter. The luciferase expression from plaP_(E1–5)_-Luc, which contains all five E-Boxes, was increased most dramatically. We further exploited CHIP-PCR to examine the direct binding of N-Myc to the predicted sites using the primer sets shown in Fig. 4c, with primers 1–3 designed to amplify the sequence containing the indicated E-boxes and primer 4 designed as a negative control. As shown in Fig. 4d, primers 1, 2 and 3 successfully amplified their corresponding PCR products from the DNAs pulled down by α-N-Myc antibody. In contrast, IgG pulldown and primer 4 did not yield any PCR product, confirming that N-Myc specifically bind to the examined E-boxes.

### N-Myc regulates endogenous expression of PLAGL2

**Fig. 5:**
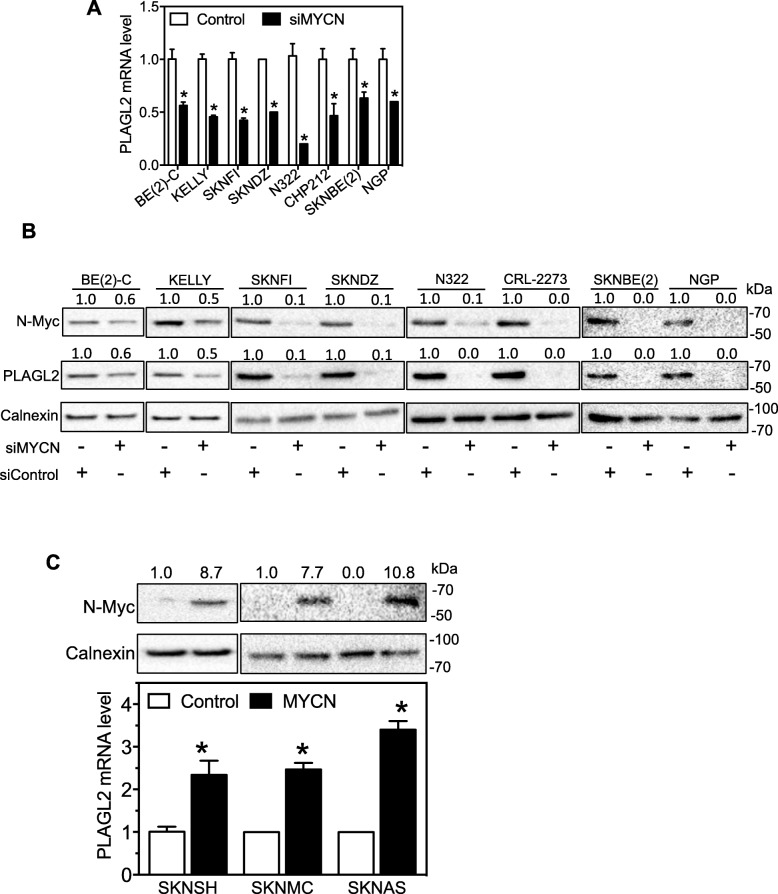
MYCN regulates endogenous expression of PLAGL2 in neuroblastoma cells. **a-b** Effect of MYCN knockdown on PLAGL2 mRNA and protein levels. Cells were transfected with siMYCN or siControl at 25 nM for 2 days. PLAGL2 mRNA (**a**) and protein (**b**) levels were detected as above. **c** Effect of MYCN overexpression on PLAGL2 expression in MYCN non-amplified neuroblastoma cells. Cells were transfected with a MYCN over-expression vector or a control vector for 2 days. The levels of N-Myc protein and PLAGL2 mRNA were detected as above. *, *p* < 0.05, comparing to control. Protein levels in the Western blot images were quantified as above

We next investigated whether N-Myc regulate endogenous expression of PLAGL2 in a panel of neuroblastoma cell lines. As shown in Fig. 5a, siMYCN significantly decreased PLAGL2 mRNA levels in all eight cell lines. Correspondingly, PLAGL2 protein levels were also dramatically decreased by siMYCN (Fig. 5b). In addition, over-expression of MYCN by a MYCN expression vector in three MYCN non-amplified cell lines dramatically increased PLAGL2 mRNA expression (Fig. 5c), further demonstrating the transcription activation of PLAGL2 by N-Myc.

Altogether, the above results indicate that miR-506-3p, PLAGL2 and MYCN form an interplay network in neuroblastoma cells.

### RAs upregulate miR-506-3p expression and downregulate the expression of both PLAGL2 and MYCN

**Fig. 6:**
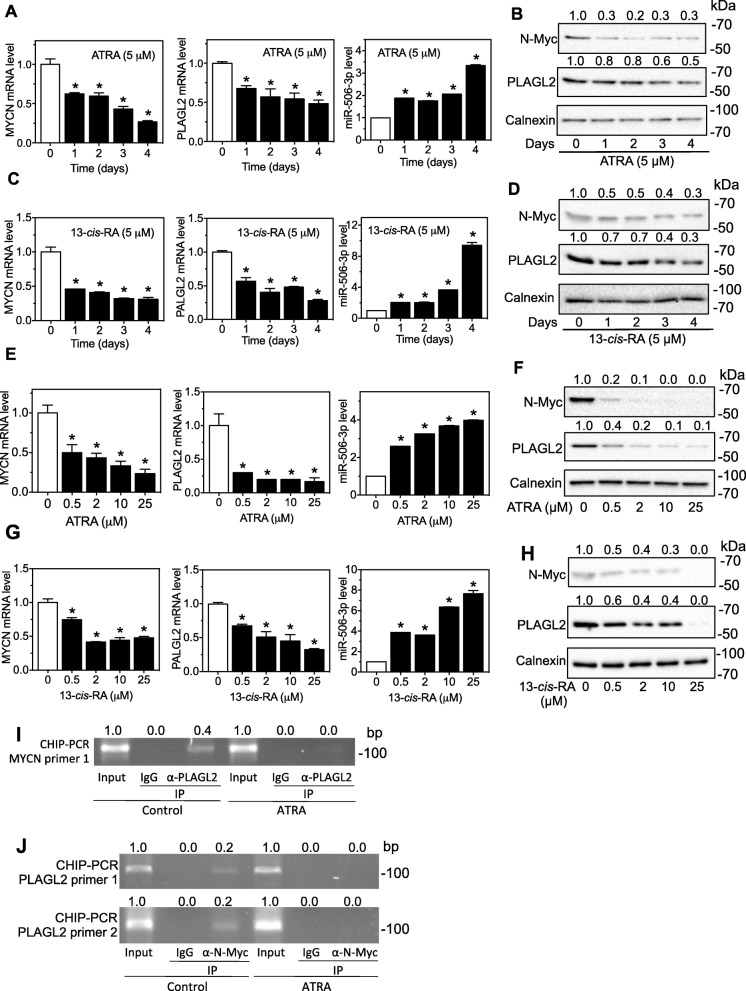
ATRA and 13-*cis*-RA downregulate expressions of MYCN and PLAGL2 in BE(2)-C cells. **a-d** Time-dependent effect of ATRA (**a-b**) and 13-*cis*-RA (**c-d**) on MYCN, PLAGL2 and miR-506-3p expressions. Cells were treated with RAs (5 μM) for up to 4 days. Shown are the RNA (**a**-**c**) and protein (**b**, **d**) levels of the indicated molecules. The data were analyzed as above. **e-h** Dose-dependent effect of ATRA (**e-f**) and 13-*cis*-RA treatments (**g-h**) on MYCN, PLAGL2 and miR-506-3p expression. Cells were treated with RAs at different concentrations for 4 days. The RNA (**e**, **g**) and protein (**f**, **h**) levels were detected as above. **i-j** CHIP-PCR assays under the treatment with ATRA. Cells were treated with ATRA (5 μM) or carrier DMSO (Control) for 4 days, and CHIP-PCR was performed as above. Protein levels in the Western blot images were quantified as above. **i**, PLAGL2 protein binding to its DNA-binding site in the promoter region of MYCN; **j**, N-Myc protein binding to its DNA-binding site in the promoter region of PLAGL2. Shown are the representative PCR results and quantifications

Differentiation agents RAs have been used for neuroblastoma therapy for decades [34, 35]. The mechanism by which RAs induce neuroblastoma cell differentiation, however, has not been fully elucidated. Here we investigated the possible involvements of the miR-506-3p, PLAGL2 and MYCN in RA signaling. As shown in Fig. 6a-d, both ATRA and 13-*cis*-RA (5 μM) significantly decreased the expression of MYCN and PLAGL2 at mRNA and protein levels, and significantly increased the expression of miR-506-3p, in a time-dependent manner. In addition, both RAs also showed a dose-dependent effect on MYCN, PLAGL2 and miR-506-3p expressions (Fig. 6e-h). Correspondingly, CHIP-PCR analysis showed that ATRA treatment abolished the PLAGL2 binding to MYCN promoter comparing to Control (Fig. 6i), further demonstrating the dramatic depletion of PLAGL2 protein expression by ATRA treatment. Similar results were observed with the binding of N-Myc to the E-boxes in the PLAGL2 promoter (Fig. 6j), confirming the dramatic depletion of N-Myc protein expression by ATRA. These results suggest the involvement of miR-506-3p, PLAGL2 and MYCN in mediating the differentiation-inducing functions of RAs in neuroblastoma cells.

### Knockdown of PLAGL2 induces differentiation, reduces survival and proliferation of neuroblastoma cells

**Fig. 7:**
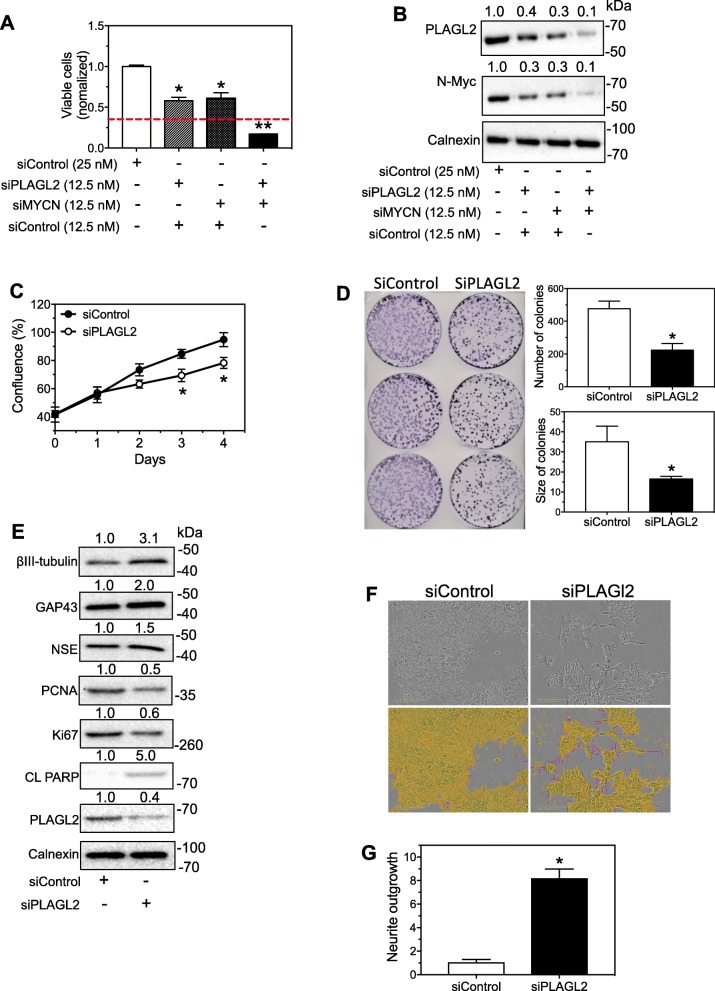
PLAGL2 regulates neuroblastoma cell survival, proliferation and differentiation. **a** Effect of siPLAG2 and siMYCN on viability of BE(2)-C cells. Cells were transfected with the indicated siRNAs or control oligo (25 nM) for 4 days, and cell viability was measured. Dashed line indicates the predicated additive effect of combined siPLAGL2 and siMYCN treatment. *, *p* < 0.05 comparing to control; **, *p* < 0.05 comparing to siPLAGL2 or siMYCN single treatment. **b** Western blots of PLAGL2 and N-Myc proteins under the same treatments as above confirmed the depletion of the protein expressions by siRNAs. **c** Time-course of cell confluence change of BE(2)-C cells after siPLAGL2 transfection. Cells were transfected with 25 nM siPLAGL2 or siControl, and cell confluence was measured using IncuCyte Zoom. *, *p* < 0.05, comparing to siControl at the same time point. **d** Effect of PLAGL2 knockdown on colony formation of BE(2)-C cells. Cells were transfected with 25 nM siPLAGL2 or siControl, and 2000 transfected cells were seeded on 100 mm dishes and cultured for 2 weeks. Crystal violet was used to stain the colonies. Show are the representative images of the dishes. *, *p* < 0.05. **e** Effect of siPLAGL2 on the protein expression levels of cell differentiation, cell proliferation and apoptosis markers. Cells were transfected with the indicated oligo and protein levels were detected and quantified as above. **f-g** Effect of PLAGL2 knockdown on neurite outgrowth in BE(2)-C cells. Cells were transfected with 25 nM oligos for 4 days, and relative neurite lengths were quantified. Shown are representative cell images analyzed to define neurite and cell body areas (**f**) and neurite length quantifications (**g**)

The cellular functions of PLAGL2 in neuroblastoma cells have not been fully defined previously. Here we investigated the cellular responses to the aberrant PLAGL2 expression in neuroblastoma cells. As shown in Fig. 7a, knocking down PLAGL2 significantly decreased cell survival, with the extent of decrease by siPLAGL2 being similar to that caused by siMYCN. Combined knockdown of MYCN and PLAGL2 showed robust synergistic effect, with the actual remaining cell viability of the combined treatment *(0.172 ± 0.005)* being dramatically lower than the predicted remaining viability due to additive effects (0.353). In parallel, the effect of the above treatments on PLAGL2 and MYCN protein expressions were confirmed (Fig. 7b). Due to the semi-quantification nature of the Western blotting approach, the synergistic effect at the protein level was not determined. In addition, Fig. 7c shows that siPLAGL2 reduced cell proliferation rate comparing to siControl, as measured by cell confluence change over time. Figure 7d further shows that siPLAGL2 decreased colony formation of BE(2)-C cells relative to siControl. Furthermore, siPLAGL2 increased expression of neuronal differentiation markers βIII-tubulin, growth associated protein 43 (GAP43) and neuron specific enolase (NSE) (Fig. 7e), indicating cell differentiation is induced. Correspondingly, it decreased expressions of cell proliferation markers proliferating cell nuclear antigen (PCNA) and Ki67, and increased expression of apoptosis marker cleaved Poly (ADP-ribose) polymerase (CL PARP) (Fig. 7e). We further examined the effect of PLAGL2 knockdown on neurite outgrowth in BE(2)-C cells. As shown in Fig. 7f-g, siPLAGL2 dramatically and significantly induced neurite outgrowth comparing to control oligo. These results altogether support the function of PLAGL2 in regulating neuroblastoma cell differentiation and proliferation.

### High tumor PLAGL2 mRNA levels are correlated with high tumor MYCN mRNA levels and poor survival of neuroblastoma patients

**Fig. 8:**
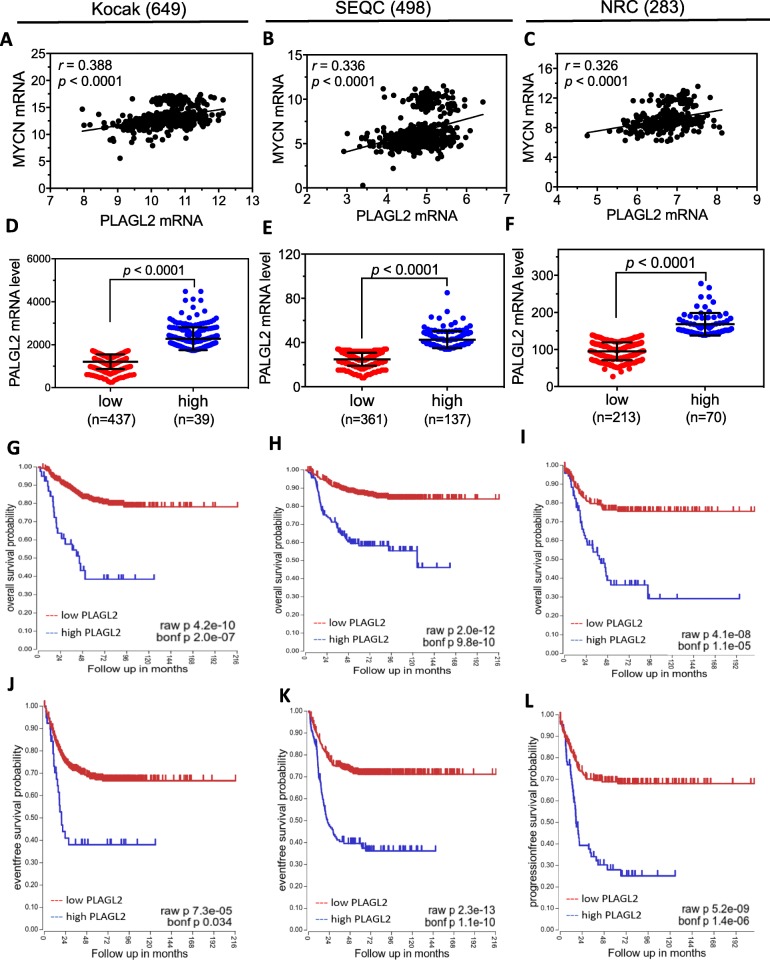
Correlation of tumor PLAGL2 mRNA levels with tumor MYCN mRNA levels and patient survival in neuroblastoma patients. (**a-c**) The correlation of tumor PLAGL2 and MYCN mRNA expression levels in the indicated three public neuroblastoma datasets, Kocak (**a**), SEQC (**b**), and NRC dataset (**c**). Shown in each graph is the correlation coefficient (*r*) and *p* values calculated based on Pearson correlation. **d-f**, Patients in the three datasets were grouped into low and high groups based on the tumor mRNA levels of PLAGL2. **g-l** Comparison of Kaplan-Meier overall (**g**-**i**) and recurrence-free (**j**-**l**) survival curves between the low and high PLAGL2 groups in each dataset

To evaluate the clinical relevance of PLAGL2 in neuroblastoma patients, we examined the correlation of PLAGL2 mRNA expression with MYCN mRNA levels in neuroblastoma patients as well as its correlation with patient survival based on published neuroblastoma patient datasets [31]. As shown in Fig. 8a-c, significant positive correlation between PLAGL2 and MYCN mRNA levels were observed in all three datasets, suggesting that the positive regulatory loop formed between PLAGL2 and MYCN play a critical role in controlling the expression of each other in neuroblastoma tumors. To examine the correlation of tumor PLAGL2 mRNA levels with neuroblastoma patient survival, the patients were classified by PLAGL2 mRNA levels into high or low PLAGL2 groups in each dataset (Fig. 8d-f). Patient survival analysis indicates that both the overall (Fig. 8g-i) and event-free survivals (Fig. 8j-l) in the high PLAGL2 groups are significantly lower than those in the low groups based on both the raw and adjusted Bonferroni *p* values in all three datasets, suggesting that elevated PLAGL2 expression is an important mechanism to drive the poor prognosis of neuroblastoma patients.

## Discussion

**Fig. 9:**
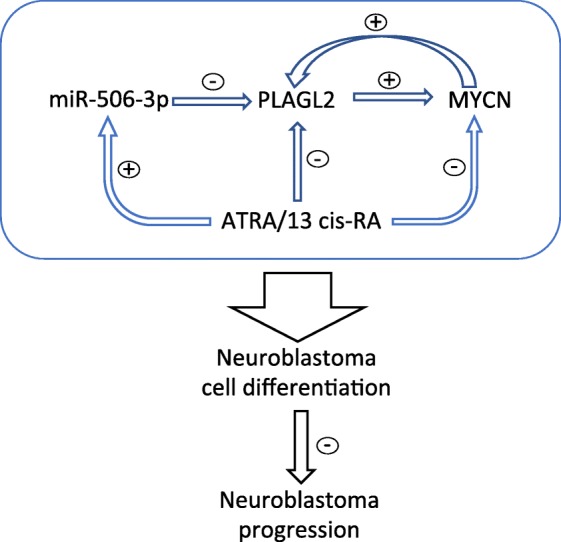
Schematic summary of the interplay between miR-506-3p, PLAGL2, MYCN and RAs. PLAGL2 and MYCN form a positive regulatory loop, which promotes the transcriptional expression of each other. miR-506-3p acts upstream of the loop and directly targets PLAGL2 and inhibits PLAGL2 expression, which subsequently lead to reduced expression of MYCN. ATRA and 13-*cis*-RA up-regulate miR-506-3p expression and down-regulate the expression of PLAGL2 and MYCN. The four-molecule interplay network plays a role in modulating neuroblastoma cell differentiation, and the imbalance of this interplay may contribute to the progression of neuroblastoma in patients

In this study, we characterized a novel interaction network involved in regulating neuroblastoma cell differentiation and potentially determining the clinical prognosis of neuroblastoma (Fig. 9). The highlight of our finding is that we demonstrate for the first time that PLAGL2, a TF that has previously been recognized as an important regulator of cell fate [36], plays a key role in modulating neroblastoma cell differentiation via interacting with three well-recognized differentiation-regulating molecules of neuroblastoma cells, MYCN, RA and miR-506-3p [17, 20, 37, 38]*.* In addition, we found that RAs regulate expressions of all the other three molecules in the network, indicating that this interplay network is a pathway mediating the therapeutic mechanism of RAs.

PLAGL2 belongs to the PLAG family of C2H2 zinc finger TFs [39, 40]. Two members in the family, PLAG1 and PLAGL2, were found to be cell growth promoting and oncogenic in several cancer cell types [41–44]. More relevant to our current study, Zheng et al. found that enforced PLAGL2 expression in neuronal stem cells and glioma initiating cells strongly impeded cell differentiation [36]. This is consistent with our findings showing that knocking down PLAGL2 expression induced neuroblastoma cell differentiation and inhibited cell proliferation. Together these findings strongly support that PLAGL2 plays a critical role in maintaining the proliferative and undifferentiated status of cells of neuronal origin, including neuroblastoma cells. In the investigation of the mechanism that mediates such function of PLAGL2, Zheng et al. showed that PLAGL2 impeded differentiation of neural stem cells and glioma initiating cells through modulating Wnt signaling [36]. Here we demonstrate for the first time that PLAGL2 directly regulates the transcription of MYCN, the well-known key regulator of neuroblastoma cell differentiation, disclosing a previously unknown mechanism underlying the differentiation-regulating function of PLAGL2. Given the demonstrated oncogenic relevance of MYCN in many types of cancers [45], whether the PLAGL2-MYCN axis is also a key player in modulating cell differentiation and proliferation in other cancer types is certainly an interesting question to address in the future. Additional transcriptional targets of PLAGL2 (e.g., MPL and ASCL2) were found in other cancer cell types [46, 47]. Given the complexity of the PLAGL2 transcriptome indicated in the published studies, it is reasonable to speculate that MYCN is not the sole transcription target that mediates the function of PLAGL2 in neuroblastoma cells. Moreover, a recent study reported a function of PLAGL2 other than acting as a TF [48], which showed that PLAGL2 modulates the stability of pirh2 through direct binding to pirh2 [48]. Also interestingly, the 3’UTR of PLAGL2 was found to be independently overexpressed in colorectal cancer cells, and has an independent function in regulating C-Myc and CD44 expression and in promoting cell proliferation and tumor growth [49]. Whether these non-TF functions of PLAGL2 play a role in modulating neuroblastoma cell fate certainly warrants future investigation.

We discovered that PLAGL2 expression is regulated by MYCN at the transcription level in neuroblastoma cells. As a key regulator of neurblastoma cell fate, whether PLAGL2 transcription is regulated by other TFs, especially TFs that are relevant in neuroblastoma tumorigenesis, is certainly a key question that needs to be addressed. PLAGL2 expression has also been shown to be regulated at the translational and post-translational levels. One mechanism is through increasing the stability of PLAGL2 mRNA, which is reported by Su et al [49]; this study showed that the HuR protein stabilized PLAGL2 mRNA by binding to its 3’UTR, and subsequently caused an increase in PLAGL2 protein expression [49]. The regulation of PLAGL2 expression by miRNAs has also been reported. For example, miR-214 was found to target the 3’UTR of PLAGL2 mRNA and down-regulated PLAGL2 expression in neuroblastoma cells [50]. miR-449a, miR-486-5p and let-7 were found to target the 3’UTR of PLAGL2 mRNA in other cancer cell types [46, 49, 51, 52]. Here we identified for the first time PLAGL2 as a direct target of miR-506-3p in neuroblastoma cells, expanding the miRNA regulatome of PLAGL2 expression. Additional mechanisms that regulate PLAGL2 expression and functions, including gene amplification and DNA insertion [44, 49], post-translational modifications of PLAGL2 protein [53, 54] and regulation of PLAGL2 activity by PLAGL2-binding proteins [55], have been reported in other cell types but have not been investigated in neuroblastoma. Overall, the molecular mechanisms that regulate PLAGL2 expression and function have been demonstrated to be complex, and future effort is certainly needed to fully dissect the molecular network involved in modulating PLAGL2 expression and functions in neuroblastoma.

Another interesting finding in our study is that RAs up-regulate miR-506-3p expression and down-regulate both MYCN and PLAGL2 expressions. While the down-regulation of MYCN by RAs was reported previously [56, 57], we are the first to define the regulation of PLAGL2 and miR-506-3p expressions by RAs. Although RAs have been identified as differentiation agents of cancer cells since 1970s [58–66] and have been used to treat neuroblastoma for decades, the molecular mechanisms underlying such function are still poorly understood. RAs are thought to regulate gene transcriptions by binding to RA receptors RAR and RXR, which are TFs that regulate gene expression by binding to the Retinoic Acid Response Elements (RARE) in the target genes [56]. However, their transcriptional targetome and the downstream signaling involved in regulating neuroblastoma cell differentiation are far from elucidated [37]. Here we found that RA treatments altered expression of all other three molecules in our identified interplay network, suggesting this is at least one of the signaling pathways mediating differentiation-inducing and therapeutic activity of RAs.

## Conclusions

In summary, we identified a novel four-molecule interplay network that plays a key role in determining neuroblastoma progression and in mediating the therapeutic function of retinoic acid. This interplay provides a possible mean to develop novel strategies to target the MYCN pathway for treating neuroblastoma. In addition, given the demonstrated oncogenic relevance of MYCN in many types of cancers, we expect that further characterization of this network in other cancer types may lead to the development of novel diagnostic and therapeutic approaches to a broad spectrum of cancers. In the future, mechanisms of interactions between the molecules in the network require further characterization in order to eventually serve the purpose of cancer diagnosis and therapy. In addition, the mechanisms of action of PLAGL2, as a new key player in regulating neuroblastoma cell fate, warrant further investigation.

## Supplementary information

**Additional file 1: Table S1.** List of primers used in qPCR and CHIP-PCR.

**Additional file 2: Table S2.** Genetic backgrounds of neuroblastoma cell lines used in this study.

**Additional file 3: Table S3.** The predicted target sites of miR-506-3p in the 3’UTRs of PLAGL2 and CREB3L2 mRNAs.

**Additional file 4: Table S4.** Changes in mRNA expression of PLAGL2 and CREB3L2 induced by miR-506-3p mimic detected in the gene expression microarray analysis.

## Data Availability

The datasets used in the current study are available from the corresponding author on reasonable request.

## Abbreviations

13-cis RA: 13-*cis* retinoic acid
3’UTR: 3′ untranslated region
ALK: Anaplastic lymphoma kinase
ATRA: All-*trans* retinoic acid
CDS: Coding sequence
CHIP-PCR: Chromatin immunoprecipitation-polymerase chain reaction
CL PARP: Cleaved Poly (ADP-ribose) polymerase
CREB3L2: CAMP Responsive Element Binding Protein 3 Like 2
GAP43: Growth Associated Protein 43
IPA: Ingenuity Pathway Analysis
miRNAs: microRNAs
NSE: Neuron-Specific Enolase
Oligo: Oligonucleotides
PCR: Polymerase chain reaction
PCNA: Proliferating cell nuclear antigen
PLAGL2: Pleiomorphic adenoma gene like-2
qPCR: quantitative PCR
RA: Retinoic acid
TF: Transcription factor
WT: Wildtype
α-N-Myc: anti-N-Myc antibody
α-PLAGL2: anti-PLAGL2 antibody
