# Supplementary material for: The PLAGL2/MYCN/miR-506-3p interplay regulates neuroblastoma cell fate and associates with neuroblastoma progression
Source: J Exp Clin Cancer Res. 2020 Feb 22;39:41. doi: 10.1186/s13046-020-1531-2 (PMC7036248; doi:10.1186/s13046-020-1531-2)
Supplement: Supplementary file 1 — Additional file 1: Table S1. List of primers used in qPCR and CHIP-PCR. [file 13046_2020_1531_MOESM1_ESM.pdf]

Table S1

Primer name	Primer sequence
GAPDH-qPCR-F	GGAGGTGAAGGTCGGAGTC
GAPDH-qPCR-R	GAAGATGGTGATGGGATTTC
MYCN-qPCR-F	CACAAGGCCCTCAGTACCTC
MYCN-qPCR-R	ACCACGTCGATTTCTTCCTC
PLAGL2-qPCR-F	CAGAGACCATATAGCTGCCC
PLAGL2-qPCR-R	CCTTGCGGTGAAACATCTTATC
CREB3L2-qPCR-F	ATGTACCACACGCACTTCTC
CREB3L2-qPCR-R	CCTCCATTGACACACTCTTCTC
CHIP-PCR MYCN primer 1F	AACACGCAGTCAAAGCGGGGG
CHIP-PCR MYCN primer 1R	CCCACCCTGCGAGCCTGGCAAT
CHIP-PCR MYCN primer 2F	ATCCTCAAACGATGCCTTCC
CHIP-PCR MYCN primer 2R	CGCCTCGCTCTTTATCTTCTT
CHIP-PCR PLAGL2 primer 1F	GACATCAAGTCCTGCCCCACTAACTCC
CHIP-PCR PLAGL2 primer 1R	TTGCAAGGGCACAGAGGGGCC
CHIP-PCR PLAGL2 primer 2F	CTCTGAGGTTCTCTCCTGGACCTGTG
CHIP-PCR PLAGL2 primer 2R	CCCTGCATGGGTAAGGCCTCCC
CHIP-PCR PLAGL2 primer 3F	GTCCCAGGAGCCCGCAGGCATCC
CHIP-PCR PLAGL2 primer 3R	GCGCTCGCGTCCCTCCTTCC
CHIP-PCR PLAGL2 primer 4F	ACCATAGCTAGCCAGTCATTTC
CHIP-PCR PLAGL2 primer 4R	GGGTACTGAGTGCAGGATAAAG

List of primers used in pPCR and CHIP-PCR. Shown are the primer name and the nucleotide sequence of each primer.
