# Supplementary material for: The PLAGL2/MYCN/miR-506-3p interplay regulates neuroblastoma cell fate and associates with neuroblastoma progression
Source: J Exp Clin Cancer Res. 2020 Feb 22;39:41. doi: 10.1186/s13046-020-1531-2 (PMC7036248; doi:10.1186/s13046-020-1531-2)
Supplement: Supplementary file 2 — Additional file 2: Table S2. Genetic backgrounds of neuroblastoma cell lines used in this study. [file 13046_2020_1531_MOESM2_ESM.pdf]

Table S2

Cell line	Age	Gender	Stage	Chr 1p alteration	Chr 17 alteration	MYCN ampl.
BE(2)-C	2	male	4	Y	Y	Y
SKNDZ	2	female	4	Y	Y	N
KELLY	unk	unk	unk	unk	unk	Y
CHLA-90	8.5	male	4	unk	unk	N
SKNFI	11	male	unk	unk	unk	N
NGP	2.6	male	unk	Y	N	Y
IMR32	1.1	male	unk	Y	N	Y
N322	unk	unk	unk	unk	unk	Y
SKNBE(2)	2	male	4	Y	Y	Y
CHP212	1.8	unk	unk	unk	unk	Y
SKNMC	1	female	4	Y	Y	N
SKNAS	8	female	4	Y	N	N
MCIXC	1	female	4	unk	unk	N
SKNSH	4	male	4	Y	N	N

Genetic backgrounds of neuroblastoma cell lines used in this study. Shown are the name of the cell line, age and gender of the patient, stage of the tumor from which the cell line was derived, chromosome 1p and 17 alterations, and MYCN gene amplification status. unk, unknown; Chr, Chromosome; ampl, amplification; Y, yes; N, no.
