# Supplementary material for: The PLAGL2/MYCN/miR-506-3p interplay regulates neuroblastoma cell fate and associates with neuroblastoma progression
Source: J Exp Clin Cancer Res. 2020 Feb 22;39:41. doi: 10.1186/s13046-020-1531-2 (PMC7036248; doi:10.1186/s13046-020-1531-2)
Supplement: Supplementary file 3 — Additional file 3: Table S3. The predicted target sites of miR-506-3p in the 3’UTRs of PLAGL2 and CREB3L2 mRNAs. [file 13046_2020_1531_MOESM3_ESM.pdf]

Table S3

<table>
<tr><th></th><th>RNA Sequence</th><th>Seed Position</th><th>Context</th><th>P_{CT}</th></tr>
<tr><td>PLAGL2 3’ UTR

miR-506-3p</td><td>5' UGUAAUAAGAAACUUUGCCUUAG 3′
|||| |||||||
3' AGAUGAGUCUUCCC-ACGGAAUG 5′</td><td>3355-3361</td><td>-0.13</td><td>0.83</td></tr>
<tr><td>CREB3L2 3’ UTR

miR-506-3p</td><td>5′ ACAUGCUCAUUUG—UGCCUUAA 3′
||||| |||||||
3′ AGAUGAGUCUUCCCACGGAAUG 5′</td><td>4175-4182</td><td>-0.08</td><td><0.1</td></tr>
</table>

The predicted target sites of miR-506-3p in the 3’ UTRs of PLAGL2 and CREB3L2 mRNAs. Shown are the predicted interactions between the target sites in the 3’UTR and miR-506-3p with the seed sequences underlined, the seed sequence position, as well as the Context and P_{CT} scores calculated by TargetScan.
