# Supplementary material for: The PLAGL2/MYCN/miR-506-3p interplay regulates neuroblastoma cell fate and associates with neuroblastoma progression
Source: J Exp Clin Cancer Res. 2020 Feb 22;39:41. doi: 10.1186/s13046-020-1531-2 (PMC7036248; doi:10.1186/s13046-020-1531-2)
Supplement: Supplementary file 4 — Additional file 4: Table S4. Changes in mRNA expression of PLAGL2 and CREB3L2 induced by miR-506-3p mimic detected in the gene expression microarray analysis. [file 13046_2020_1531_MOESM4_ESM.pdf]

Table S4

Gene name	Ratio of mRNA level (miR-506-3p/control)	
	24 h	72 h
PLAGL2	0.470	0.785
CREB3L2	0.514	0.455

Changes in mRNA expression of PLAGL2 and CREB3L2 induced by miR-506-3p mimic detected in the gene expression microarray analysis. Shown are the gene name and the fold change of mRNA expression levels in terms of miR-506-3p vs. control treatment following 24 h and 72 h of transfection with miR-506-3p mimic in BE(2)-C cells.
